# SARCOPENIA AND GASTROINTESTINAL CANCER: NUTRITIONAL APPROACH FOCUSING ON CURCUMIN SUPPLEMENTATION

**DOI:** 10.1590/S0004-2803.24612024-068

**Published:** 2025-04-04

**Authors:** Pamela S de ALMEIDA, Katia BARÃO, Nora M FORONES

**Affiliations:** * Universidade Federal de São Paulo, Escola Paulista de Medicina, Departamento de Medicina, Divisão de Gastroenterologia, São Paulo, SP, Brasil.

**Keywords:** Sarcopenia, cancer, curcumin, nutritional status, sarcopenia, câncer, curcumina, estado nutricional

## Abstract

Sarcopenia is a syndrome characterized by decreased strength, quantity and/or quality of skeletal muscle mass. When associated with cancer, it correlates with poorer clinical outcomes. Cancers of the gastrointestinal tract, prevalent globally and in Brazil, are associated with a greater nutritional risk. Early detection and intervention for nutritional risks are critical in this population. Recent studies on turmeric/curcumin have demonstrated beneficial effects in cancer patients. Specifically, curcumin have shown promise in reducing muscle depletion, oxidative stress, and improving strength and fatigue, factors related to sarcopenia. This review aims to elucidate sarcopenia and sarcopenia secondary to cancer, emphasizing nutritional management and the role of curcumin supplementation. Effective cancer management, whether with or without sarcopenia, demands comprehensive public health strategies and multimodal interventions within healthcare institutions. Nutrition is pivotal across the cancer care journey, encompassing screening, guidance, and provision of nutrients that support maintaining or recovering body composition. Curcumin supplementation emerges as a potential adjuvant to the standard cancer treatment and sarcopenia management. Nevertheless, further clinical studies are warranted to substantiate these findings.

## HIGHLIGHTS

• Sarcopenia is a syndrome characterized by decreased strength, quantity and/or quality of skeletal muscle mass.

• Sarcopenia when associated with cancer, it correlates with poorer clinical outcomes.

• Curcumin has shown promise in reducing muscle depletion, oxidative stress, and improving strength and fatigue, factors related to sarcopenia.

• Curcumin supplementation emerges as a potential adjuvant to the standard cancer treatment and sarcopenia management.

## RESUMO

A sarcopenia é uma síndrome caracterizada pela diminuição da força, quantidade e/ou qualidade da massa muscular esquelética. Quando associada ao câncer, ela correlaciona-se a piores resultados clínicos. Os cânceres do trato gastrointestinal, prevalentes globalmente e no Brasil, estão associados a um maior risco nutricional. É fundamental que haja detecção e intervenção nutricional precoces nessa população. Estudos recentes sobre cúrcuma/curcumina demonstraram efeitos benéficos em pacientes com câncer. Especificamente, a curcumina mostrou-se promissora na redução da depleção muscular, estresse oxidativo e melhora da força e fadiga, fatores relacionados à sarcopenia. Esta revisão visa elucidar a sarcopenia e a sarcopenia secundária ao câncer, enfatizando o manejo nutricional e o papel da suplementação de curcumina. O manejo eficaz do câncer, com ou sem sarcopenia, exige estratégias abrangentes de saúde pública e intervenções multimodais dentro das instituições de saúde. A nutrição é fundamental em toda a jornada de tratamento do câncer, abrangendo triagem, orientação e fornecimento de nutrientes que apoiam a manutenção ou recuperação da composição corporal. A suplementação de curcumina surge como um potencial adjuvante ao tratamento padrão contra o câncer e no manejo da sarcopenia. No entanto, mais estudos clínicos são necessários para consolidar essas descobertas.

## INTRODUCTION

The European Working Group on Sarcopenia in Older People (EWGSOP) defined sarcopenia as a syndrome characterized by progressive and generalized loss of skeletal muscle mass and strength, which can result in loss of function, worsening of quality of life, and death^1^. In 2019, the EWGSOP2 updated this definition, emphasizing that the decrease in muscle strength is considered the main characteristic of sarcopenia and a parameter for its diagnosis, which also considers not only the quantity but also the quality of muscle^2^. 

Sarcopenia is considered primary when it is originally associated with the aging process; and secondary when associated with physical inactivity, insufficient protein-energy intake, and chronic diseases, such as cancer. When it affects overweight and obese individuals, it is called Sarcopenic obesity^2^ ^-^ ^4^.

Cancer is the second leading cause of early death around the world, both in wealthy and developing countries, accounting for 4.5 million (29.8%) deaths. This is due to demographic changes and lifestyle habits associated with urbanization, such as inappropriate eating behavior, smoking, and sedentary lifestyle, which are also associated with the most common types of cancer in Brazil and worldwide^5^ ^,^ ^6^.

Despite the evidence that premature mortality rates tend to decrease in Brazil between 2026-2030, both in men (-12.9%) and in women (-4.5%) in most Brazilian regions^7^, it is estimated that between the years 2023-2025 there will be 704 thousand new cases of cancer. Among these, the incidence rates of the most frequent cancers, excluding non-melanoma skin cancer, are: 10.5% breast, 10.2% prostate, 6.5% colorectal, and 4.6% lung cancer^5^ ^,^ ^6^.

Cancer patients can develop cachexia, a syndrome that is associated with chronic conditions or diseases in the terminal stages. Although it is still an underdiagnosed and undertreated condition^8^. Studies indicate that cachexia affects half of the patients in advanced progression, and is responsible for more than 30% of deaths among these individuals^3^ ^,^ ^4^ ^,^ ^9^ ^,^ ^10^. Its pathophysiology involves several metabolic pathways, tissues and organs, and is characterized by a set of factors that lead to intense catabolism, resulting in progressive loss of skeletal muscle mass and adipose tissue^4^ ^,^ ^9^. Unlike cachexia, sarcopenia occurs independently of weight loss and can be considered a component of cachexia^4^ ^,^ ^11^. Even though cancer is a highly prevalent disease and its correlation with sarcopenia is associated with worse clinical outcomes, there are still few studies regarding the prevalence of sarcopenia in these individuals^3^ ^,^ ^11^ ^-^ ^14^. 

Health bodies around the world emphasize the importance of healthy habits, including the practice of physical activity and a protective diet, creating a metabolic state capable of genetically and epigenetically modulating pathways involved in carcinogenesis, thus preventing or aiding in the treatment of cancer, avoiding the worsening of nutritional status and, consequently, the emergence of diseases associated with malnutrition^9^ ^,^ ^10^ ^,^ ^15^ ^-^ ^21^.

Genetic modulation is possible thanks to specific nutrients or isolated food components rich in bioactive compounds, such as organosulfur compounds, flavonoids, polyphenols, isothiocyanates, among others, which have anti-inflammatory, antitumor, antioxidant and chemoprotective functions, and are present in crucifers, vegetables, nuts, citrus fruits, garlic, tomatoes, grapes, saffron and many other foods^5^ ^,^ ^15^ ^,^ ^17^.

Polyphenols are bioactive compounds much studied by the scientific community, especially curcumin, a yellow polyphenol obtained from the rhizomes (underground stems) of Curcuma longa, a member of the ginger family, also popularly called saffron (Brazil), haldi (India), ukon (Japan). It has been widely used for millennia in Asian medicine and Ayurveda due to its coloring, aromatic, stimulating properties and also an as potent antioxidant and anti-inflammatory^22^ ^-^ ^24^. Despite its potential, it has low bioavailability, which has been attenuated by pharmaceutical technologies, making its applicability in clinical trials more viable and effective^25^ ^-^ ^31^.

In recent decades, studies involving curcumin and/or other curcuminoids have grown exponentially, and several works at experimental and clinical levels show beneficial effects in cancer patients. Its effectiveness includes the management of inflammation, chemoprevention, and as a complementary treatment to conventional treatment, as it supposedly acts as an anticarcinogenic agent for several tumors^24^ ^,^ ^32^ ^-^ ^37^. Furthermore, curcumin and bioavailable curcumin were able to decrease muscle depletion and oxidative stress, and improve strength and fatigue, factors related to sarcopenia^38^ ^-^ ^41^.

This review aims to provide an understanding of sarcopenia and sarcopenia secondary to cancer and their nutritional management, with a focus on curcumin supplementation.

## METHODS

A literature scope review was carried out, considering articles published between 2018 and 2023, using electronic databases of scientific reliability PUBMED/MEDLINE, LILACS and Embase. The descriptors in health sciences (DeCS) were used for the search: sarcopenia, cancer, curcumin, nutritional status, adding the Boolean logic AND, OR, NOT, or medical subject headings (MeSH). Original scientific articles, systematic reviews, meta-analyses, national and international reviews and guidelines in Portuguese and English were included.

### Diagnosis of sarcopenia

The EWGSOP2 suggests that some steps should be taken for the diagnosis of sarcopenia. The first is the identification of individuals at risk for sarcopenia, and for this purpose, they recommend the validated questionnaire, SARC-F, consisting of five questions regarding the ability to exert force, walk, get up from a chair or bed, climb stairs and whether the individual had falls in the last year, considering a score ≥4 as a predictor of sarcopenia^2^ ^,^ ^42^. In 2016, Barbosa-Silva et al.^43^ observed that the SARC-F has questions that only assess muscle function and not muscle mass, for this reason, they added the measurement of calf circumference to the questionnaire, making the detection of more sensitive sarcopenia. The SARC-Calf was validated in the Brazilian population, and therefore, it is recommended as a screening tool by the Brazilian Society of Geriatrics and Gerontology (BSGG)^44^.

After the screening, the next step will assess muscle strength. For this, the handgrip strength (HGS) test is performed using a dynamometer, and cutoff points <27 kgf for men and <16 kgf for women; or by the sit-to-stand test, with a cut-off point of >15 seconds 5 times, to detect probable sarcopenia. Negative cases, either in screening or in the evaluation stage, must be reassessed at another opportunity^2^.

The third step is confirmation, which can be performed by detecting low muscle quantity and/or quality, using body mass index (BMI), total skeletal muscle mass (TSMM), appendicular skeletal muscle mass (ASMM) (<20 kg for men; <15 kg for women), or by muscle cross-sectional area (mCSA); such parameters can be obtained by different evaluation methods, such as computed tomography (CT) or magnetic resonance imaging (MRI), considered gold standard methods for assessing muscle quantity and quality, or even by dual-energy x-ray absorptiometry (DEXA), bioimpedance (BIA) or even by calf circumference (CP) (<31 cm), and their respective cutoff points, which may or may not be adjusted for height^2^, weight or BMI. The choice of the method varies according to the availability and feasibility of your application^2^.

Once sarcopenia has been confirmed, it is recommended to identify its severity, through the evaluation of functionality, using one of the following tests: walking speed (≤0.8 m/s in 6 m) (recommended by the BSGG), Timed up and go (TUG) (stand up from a chair, walk 3 m, turn around, walk 3 m and sit down, in ≤20 s), 400 m walk (no completion or ≥6 min. completion), or Short Physical Performance Battery (SPPB) (≤8 points). After performing the functionality test, it is then possible to conclude the diagnosis and classify it as probable sarcopenia, sarcopenia, or severe sarcopenia^2^ ^,^ ^44^.

Spexoto et al.^45^, in a longitudinal study with 6182 individuals, aged ≥60 years, compared the definitions and cutoffs established by the EWGSOP1 and EWGSOP2, and analyzed the accuracy of the HGS and gait speed to identify the risk of mortality; and concluded that the cutoff point for decreased muscle strength of <36 kgf for men and <23 kgf for women and gait speed ≤0.8 m/s, demonstrated better accuracy and better predictive value for risk of mortality in the elderly. The new results suggest, perhaps, a more critical and careful look even in individuals who did not have HGS <27 kgf and <16 kgf for men and women respectively.

It is important to remember that there are different diagnostic criteria and cutoff points for sarcopenia established around the world, such as those defined by the International Working Group on Sarcopenia (IWGS) or by the Asian Working Group for Sarcopenia (AWGS), this variability in diagnostic criteria has a direct impact on the heterogeneity of studies published so far, making it even more difficult to determine the prevalence and incidence of sarcopenia in different populations^46^ ^-^ ^49^.

Sarcopenic Obesity (SO) is defined as the presence of excess adipose tissue (BMI >30 kg/m^2^ or high waist circumference according to ethnic cutoff points) associated with sarcopenia. SO can also affect young or middle-aged individuals, in addition, the inflammation generated by the adipose tissue itself, or the presence of other acute or chronic diseases, can aggravate the sarcopenia picture. Due to its implication in unfavorable clinical outcomes, its diagnosis and specific interventions are extremely important^50^.

Sarcopenia secondary to cancer, especially gastrointestinal tract cancers, has a prevalence of 43.68% according to Haiducu et al.^51^. However, there are no specific tools for diagnosing sarcopenia in this population, which is often also associated with malnutrition, which makes it essential to create protocols for early detection and intervention, both on an outpatient basis and during hospitalization, through the use, adaptation or associations of existing tools, further expanding the opportunities for studies in this area.

### Nutritional recommendations in cancer and secondary cancer sarcopenia

### Nutritional screening and assessment

Among the most frequent cancers are lung and colorectal cancer, in which patients are at greater nutritional risk. Other one are tumors of the head and neck and of the gastrointestinal tract (GIT) in general (esophagus, stomach, duodenum, pancreas, and liver)^6^ ^,^ ^20^ ^,^ ^42^ ^,^ ^52^. Because of this, it is essential to detect nutritional risk and intervene early in this population, with the aim of preventing the onset or evolution of cachexia and sarcopenia^20^ ^,^ ^42^ factors that directly impact the treatment, prognosis, and quality of life of the patient^12^ ^,^ ^21^ ^,^ ^53^ length of hospital stay, and consequently, hospitalization costs^20^ ^,^ ^54^ ^,^ ^55^.

National and international guidelines for nutritional therapy in cancer recommend that early screening and nutritional assessment be carried out, using some of the already validated tools such as Nutritional Risk Screening (NRS-2002), Subjective Global Assessment (SGA, or a reduced version or Patient Generated, PG-SGA), Short Form Mini Nutritional Assessment (MNA-SF) (elderly) and Malnutrition Universal Screening Tool (MUST)^16^ ^,^ ^56^.

The PG-SGA provides information on gastrointestinal symptoms, food intake, weight change, functional capacity, and physical examination, and precisely because it is a more comprehensive tool, containing essential items of nutritional assessment, it was translated, validated, and is specifically recommended for screening and evaluation of cancer patients in Brazil^57^. As a complementary to the PG-SGA, in outpatient care, the tool Global Leadership Initiative in Malnutrition (GLIM) has been extensively studied for the detection of malnutrition in individuals with cancer^58^ ^,^ ^59^, however, its application in practice is still not very common in Brazil.

After screening and nutritional assessment, it is possible to determine whether cachexia is present or not, and for the diagnosis of secondary sarcopenia, it is recommended to carry out all the steps suggested by the EWGSOP2, previously mentioned^56^ ^,^ ^57^. For aging patients with cancer, it is also recommended to assess serum levels of albumin, total cholesterol, and C-reactive protein (CRP), which may be related to malnutrition^56^.

### Energy and protein recommendations

Energy needs can be determined by indirect calorimetry, a method considered the gold standard, but if unavailable, pocket, or predictive formulas are well accepted and used in clinical practice^16^.

The European Society for Clinical Nutrition and Metabolism (ESPEN)^16^ ^,^ ^20^, the Brazilian Society of Parenteral and Enteral Nutrition (BRASPEN/SBNPE)^57^ and the Brazilian Society of Oncological Nutrition (SBNO)^56^ suggest for adult and elderly patients undergoing treatment 25-30 kcal/kg/day; for patients with cancer and cachexia or malnourished 30-35 kcal/kg/day.

It is important to pay special attention to patients with severe malnutrition or with cachexia due to the risk of refeeding syndrome, in these cases, it is recommended to offer 5-20 kcal /kg/day with slow onset and progression in the first week, along with monitoring of serum levels of phosphorus, magnesium, potassium, thiamine and blood glucose^56^ ^,^ ^57^.

The recommended energy intake for elderly with BMI <18.5 kg/m^2^, 32-38 kcal/kg/day^57^; for critical patients 15-25 kcal/kg/day (consider the metabolic stress phase)^56^; and finally, obese patients should have 20-25 kcal/kg/ideal weight/day^56^ ^,^ ^57^ or 11-14 kcal/kg/current weight/day^56^.

Specific energy recommendations for decreased muscle mass in cancer also range from 25-30 kcal/kg/day, according to Prado et al.^19^.

Regarding protein needs, in general, a high-protein diet should be considered for cancer patients with or without sarcopenia, ranging from 1.2-1.5 g/ptn/kg/day or more, and may reach up to 2.5 g/ptn/kg/day in critical phase diseases (exacerbated catabolic stress)^16^ ^,^ ^19^ ^,^ ^44^ ^,^ ^48^ ^,^ ^56^ ^-^ ^58^. 

However, a recent randomized, multicenter clinical trial, aimed to verify whether offering high protein doses (≥2.2 g/kg) to critically ill patients would improve their clinical outcomes, and from the results obtained, the researchers do not support offering high protein doses to patients on mechanical ventilation, elderly, obese, more serious, frail, malnourished or sarcopenic patients. They concluded that the supply of protein at 1.2 or 1.3 g/kg/day, according to the American and European guidelines for enteral and parenteral therapy (ASPEN and ESPEN), seems to be adequate and safe for critically ill patients. The authors suggest that further clinical studies are still needed involving burned, polytraumatized, obese, and post-surgical patients, who may benefit from high doses of protein^60^.

Therefore, the protein recommendations of 1.5 g/kg/day for cancer patients with low muscle mass suggested by Prado et al.^19^, seems to be sufficient and safe to help modulate body composition.

For individuals with non-dialytic chronic kidney disease, 0.6-0.8 g/ptn/kg/day should be considered to avoid worsening kidney function^44^.

In addition to adequate protein supply, it is important to divide it into 20-30 g per meal^19^ ^,^ ^44^.

In case of insufficient food intake, that is, <70% of nutritional needs, the beginning of oral nutrition support (ONS) using hypercaloric and/or protein supplements, as well as adaptation of food and nutritional advice according to the presence of gastrointestinal symptoms. Enteral nutritional (EN) should be considered when there is the persistence of inadequate oral food intake and functioning GIT; and in the impossibility of access to the GIT, and/or insufficient EN, consider parenteral nutritional (PN)^56^ ^,^ ^57^.

### Complementary nutritional recommendations

Omega-3 polyunsaturated fatty acids, especially Eicosapentaenoic (EPA) and Docosahexaenoic (DHA), are some of the most studied and well-defined nutrients in the literature for use in cancer patients undergoing chemotherapy treatment with sarcopenia or for its prevention, as showed to be able to contribute to the improvement of appetite and food intake, increase in lean mass and body weight, so it is recommended to use 2.0 g of omega-3 fatty acid, or more specifically 2.0-2.2 g/ EPA and 1.0-1.5 g/DHA^16^ ^,^ ^19^ ^,^ ^57^.

As previously discussed, the adequate and fractionated protein offered is extremely important for the management and prevention of malnutrition, cachexia, and sarcopenia in cancer patients, in addition, the supply of branched-chain amino acids (Leucine, Isoleucine, and Valine, also known as BCAA), however, the supply of 3 g of Leucine proved to be more efficient in promoting muscle anabolism, as well as its metabolite, β-hydroxy-β-methyl butyrate (HMB), also with the ingestion of 3 g/day together with the administration of other amino acids and never in isolation^11^ ^,^ ^16^ ^,^ ^19^ ^,^ ^44^. However, the evidence for leucine and HMB supplementation to treat or prevent sarcopenia and cachexia in cancer is still controversial^19^ ^,^ ^48^ ^,^ ^58^. Therefore, the use of those supplements is not recommended by the Brazilian nutritional therapy guideline for cancer patients^57^.

Other amino acids and their derivatives, such as glutamine, creatine, β-alanine, and carnitine have also been studied to help manage sarcopenia and/or cachexia in cancer, and although the findings published so far show certain benefits, they still show inconsistent results for to establish its standardization in clinical practice^19^ ^,^ ^58^.

The practice of physical activity, when possible, and especially resistance exercises, is fundamental in association with adequate caloric and protein intake, and should be encouraged in cancer and/or sarcopenic patients, as in this population physical activity is related to improved aerobic capacity, muscle strength, quality of life, fatigue, etc^16^ ^,^ ^19^ ^,^ ^20^ ^,^ ^48^ ^,^ ^58^.

Vitamin D is a nutrient that performs several functions in the human body, among them is its involvement in the control of cell proliferation and oncogenesis, as well as in the control of the skeletal muscle system; due to such physiological importance, evidence has associated low levels of vitamin D, <20 ng/mL, with cancer, low muscle mass and sarcopenia, thus justifying the need for its supplementation in cases of deficiency^16^ ^,^ ^57^. In cancer patients, supplementation of 600-800 IU per day of vitamin D seems to suggest benefits for the prevention and treatment of low muscle mass, avoiding the worsening of the nutritional status^19^.

In addition to omega-3 and vitamin D, considered anti-inflammatory nutrients, other substances with the same characteristic are patched, such as the polyphenols found in grapes (resveratrol) and turmeric (curcumin), among other bioactive compounds, as they are able to reduce the inflammatory response, through the reduction of nuclear factor kappa-β (NF-kβ) signaling and oxidative stress, involved in muscle catabolism. However, there are still no established doses for supplementation in clinical practice^19^ ^,^ ^36^ ^,^ ^37^ ^,^ ^58^ ^,^ ^61^.

The recommendations for the management of sarcopenia/cachexia secondary to cancer also include pharmacological intervention in cases individually evaluated by the oncologist, who may benefit from the use of anti-inflammatory substances, progestins, antipsychotics, orexigenic, among others, such as corticosteroids, medroxyprogesterone or megestrol acetate, olanzapine, and anamorelin (ghrelin receptor agonist)^9^ ^,^ ^16^ ^,^ ^62^.

Cancer treatment per se is complex and multifactorial, and when linked to cachexia-anorexia syndrome and/or sarcopenia, its management becomes even more difficult, thus multimodal intervention, that is, with two or more modalities that aim to improve a common objective, can generate positive results such as the decrease in muscle catabolism, improvement in gastrointestinal symptoms, psychological improvement, among other factors involved in this condition^9^ ^,^ ^16^ ^,^ ^62^.

### Curcumin and sarcopenia

The first works with curcumin, published in scientific databases, date back to 1949, but only gained strength in the 1970s. In 1980 the first findings related to curcumin supplementation and cancer. Later, in 1992, the first study was published involving curcumin and endothelial smooth muscle cells^63^, and finally, in 1999, researchers sought to find an effective treatment to stimulate the repair of skeletal muscle tissue with the use of systemic curcumin in live models^64^. From then on, published works grew exponentially up to the present day.

Recently, some studies that used curcumin, as well as its different bioavailable formulations, for the management of sarcopenia have been published and have shown less muscle depletion, as well as its restoration, improvement in physical performance, increase in handgrip strength, and therefore, improves sarcopenia, as described hereafter.

Receno et al.^39^, used living models of rats at an advanced age and verified the impact of prolonged curcumin supplementation on muscle mass, functionality, and levels of oxidative stress. Rats were divided into three groups, CON (control), which was fed *ad libitum*; CUR was also fed *ad libitum*, but received curcumin; and finally, the group of paired PAIR rats received similar food and supplementation to CUR group, but a week later and in smaller quantities. Supplementation lasted 4 months. The researchers observed that PAIR rats had lower strength compared to CUR (*P*=0.040); CUR showed higher levels of nuclear factor-erythroid 2 related factor 2 (Nrf2) (master transcriptional regulatory factor of antioxidant defenses) compared to PAIR mice (*P*=0.008), as well as less oxidative damage of macromolecules; and finally, the plantar muscle mass of PAIR rats was significantly lower than that of CON and CUR rats (*P*=0.021 and *P*=0.011, respectively). Based on these findings, the researchers concluded that curcumin supplementation can contribute to the improvement of muscle mass and function, especially during a state of food restriction, and thus has potential relevance for use in specific conditions, in which food intake is compromised or insufficient.

In another experimental study, Gorza et al.^65^ used two different strains of male rats, with an initial age of 18 months, and administered curcumin or just the vehicle subcutaneously, every 6 days, for a total period of 6 months, aiming to prove the effectiveness of curcumin against selective weight loss muscle and/or strength loss induced by aging (pre-sarcopenia or sarcopenia). Effects on survival, liver toxicity, loss of muscle mass and strength, and satellite cell response and impairment were evaluated. The procedure was well tolerated, and no adverse effects were observed. As a result, they obtained a reduction in spontaneous mortality in the group treated with curcumin in both strains (*P*=0.04), and consequently, an increase of +20-35% in survival; curcumin also significantly compensated (-15%, *P*=0.04) the loss of soleus and extensor *digitorum longus* (EDL) muscle mass in one of the strains; reduced age-related decrease in specific tetanic tension (*P*<0.05), improving contractility; effects caused by stress and aging on the number, type, and size of muscle fibers were attenuated with curcumin treatment; in soleus and EDL muscles with signs of pre-sarcopenia and sarcopenia, at the cellular level, curcumin partially prevented changes in proteins and regulatory components of costamers (a cellular component of striated muscle that connects the sarcomere to the cell membrane), and positively affected the levels of satellite cells involved in myofiber maturation. Therefore, they concluded that treatment with curcumin successfully prevents the development of pre-sarcopenia and sarcopenia in rats.

Liang et al.^66^ developed and tested a type of bioavailable curcumin for the prevention of sarcopenia, called Cur-SHAP, and it is composed of stearic acid and a hydrophobic surface to which curcumin binds. Intramuscular administration is proposed and after undergoing phagocytosis by macrophages, curcumin is released into the bloodstream. Tests were performed in vitro and live mouse models; and showed good biocompatibility and excellent antioxidant and anti-inflammatory effects in in vitro tests, and improved muscular endurance, grip strength, and fat/lean mass ratio in animal models of sarcopenia treated with Cur-SHAP.

Studies in humans for the management of sarcopenia through curcumin supplementation have also been developed, such as the clinical, randomized, placebo-controlled, double-blind study, published by Varma et al.^38^, which evaluated the effectiveness of bioavailable curcumin, called Cureit^®^, in the management of sarcopenia in healthy elderly people. A total of 30 individuals were evaluated and randomly divided into two groups, which received capsules with 500 mg of placebo or Cureit^®^. Volunteers took the supplementation for 3 months and adverse events were monitored through monthly visits. The elderly supplemented with Cureit^®^ showed an improvement of 1.43% in handgrip strength (*P*<.001); a 6.08% increase in the ability to lift weight; and a trend of positive impact on the distance covered (an increase of 5.53% vs 2.29% in the placebo group) before the perception of tiredness (*P*=0.09). From the results, the group concluded that Cureit^®^ plays a significant role in the treatment of sarcopenia.

An older study, published in 2016, similar to the one cited above, also used a bioavailable curcumin formulation, called Meriva^®^, to assess its effectiveness in the management of sarcopenia in healthy elderly people. For this purpose, the volunteers were divided into 3 groups: 1- standard intervention (exercise + balanced diet); 2- standard intervention + 1 g/day Meriva^®^ and 3- standard intervention + 1 g/day Meriva^®^ + Vitamin D 800 IU/day + Vitamin D C 500 mg/day + Isoleucine 3 g/day + Carnitine 1 g/day. The duration of the study was 3 months, and the volunteers were evaluated at the beginning and the end of the protocol. The variables analyzed were handgrip strength, the ability to lift a weight, pedal, walk, and climb stairs, among others. The results show significant improvement (*P*<0.05) in all evaluated parameters in the two groups that received Meriva^®^ about the group that did not receive^67^.

Furthermore, these findings related to the improvement of sarcopenia, clinical, randomized, placebo-controlled, double-blind studies with cancer cachexia-anorexia syndrome (CACS) patients also show some benefits with supplementation of 800-2000 mg of curcumin for 8 weeks, among the results found, are a significant increase in mean muscle mass (*P*=0.03), as well as less weight loss, but with no statistically significant difference between groups (supplementation vs placebo)^68^; and they also observed a tendency towards a reduction in the decrease in handgrip strength^68^ ^,^ ^69^.

The presented results can be justified due to the potent antioxidant and anti-inflammatory action of curcumin, reducing oxidative stress, by increasing mitochondrial biogenesis, or by increasing Nrf2 and SIRT1, for example, but also attenuating the signaling of muscle catabolism pathways, in which they involve nuclear proteins such as NF-kβ, and suppressing the activity of the ubiquitin-proteasome system (UPS), one of the main systems involved in the physiological or pathological muscular atrophy process^40^ ^,^ ^61^ ^,^ ^70^ ^,^ ^71^.

The studies cited earlier in this topic have limitations and the wide variation in the types of curcumin available in the pharmaceutical industry, on the one hand, helps researchers to obtain better results regarding bioavailability and standardization for supplementation, but on the other hand, generates many heterogeneous results. to be used in more robust works with greater scientific weight. However, none of the cited studies present results of curcumin supplementation, specifically administered to cancer and sarcopenic patients, opening doors for future clinical research.

### Curcumin and gastrointestinal cancer

In the extensive literature available on turmeric and curcuminoids, the safety of using this phytochemical is almost unanimous and there are no risks, even at high doses (8-12 g/day) for a long period of use, both in healthy individuals or in the presence of diseases, including cancers, and individuals undergoing treatment with different chemotherapy drugs, however, in pregnant women, nursing mothers, and children, there are still not enough studies to guarantee the safety of use in these populations^23^ ^,^ ^72^ ^-^ ^75^.

The potential benefits of using curcumin in patients with different types of cancer can be numerous, such as anti-inflammatory, reducing the production of inflammatory cytokines, due to the inhibition of the tumor necrosis factor-α (TNF-α) signaling pathway; antitumor, by inhibiting tumor proliferation, invasion, metastasis, and angiogenesis, inducing apoptosis and/or autophagy pathways or by increasing cytotoxicity, increasing the production of reactive oxygen species (ROS) and consequently killing cancer cells; inhibiting muscle depletion signaling pathways; suppressing totipotent features of tumor cells. In addition to promoting synergism with chemotherapy drugs, enhancing their action and reducing the effects of chemotoxicity; hepatoprotection, among others^27^ ^,^ ^35^ ^,^ ^67^ ^,^ ^72^ ^,^ ^76^ ^-^ ^78^.

The metabolization of curcumin occurs mainly by the UDP-glucuronosyltransferase (UGT) enzyme, present in the intestine, but this enzyme is also found in the liver and kidneys and is responsible for the metabolization of several chemotherapeutics, including Irinotecan (camptothecin- topoisomerase inhibitor II). Because of this, there is a theoretical risk of toxicity when co-administered with curcumin by possibly inhibiting UGT. However, recently published experimental works (in vitro) show positive results of curcumin regarding its concomitant use with the chemotherapeutic Irinotecan, as attenuation of resistance to irinotecan; protective effect against the drug with a decrease in diarrhea symptoms and alterations in the intestinal mucosa^74^ ^,^ ^79^ ^,^ ^80^. And in humans, a phase I/II study evaluated the safety of administering bioavailable curcumin (curcumin complex phosphatidylcholine) concomitantly with irinotecan in patients with advanced solid tumors. Curcumin was administered in a maximum dose of 4 g, and for this amount, there was no dose-limiting toxicity. There was no increase in irinotecan-related adverse events, not even reports of grade 3 diarrhea; cases of grade 3 neutropenia occurred in 9% of the study participants, that is, a lower incidence than that demonstrated in previous studies (10 and 18%). And finally, bioavailable curcumin was not able to change the plasma concentrations of irinotecan, or its metabolite (SN-38)^81^.

Specifically in cancers of the gastrointestinal tract, studies demonstrate the synergism of curcumin concomitantly with different chemotherapy drugs such as paclitaxel, cisplatin, 5-fluorouracil (5-FU), oxaliplatin, gemcitabine, and even irinotecan and other drugs used in other types of tumor, such as doxorubicin, etoposide, vincristine and methotrexate, and with that, decreased chemoresistance, improved tumor response and reduced side effects^27^ ^,^ ^82^. Howells et al.^83^, in a randomized, controlled, phase IIa clinical trial, demonstrated that curcumin (0.5-2 g) is safe and tolerable as an adjuvant to FOLFOX (folinic acid +5-FU + oxaliplatin) as a chemotherapy treatment for patients with metastatic colorectal cancer, moreover, the group treated with curcumin and FOLFOX (CUFOX) had an increase in disease-free survival of 0.57 (95%CI: 0.24, 1.36; *P*=0.2) (median of 171 and 291 days for FOLFOX and CUFOX, respectively) and overall survival of 0.34 (95%CI: 0.14, 0.82; *P*=0.02 ) (median 200 and 502 days for FOLFOX and CUFOX, respectively). However, the work has a reduced sample size, making phase III studies necessary.

In esophageal cancer, one of the most incident types of cancer around the world, many experimental studies have demonstrated several of the previously mentioned benefits after the use of curcumin^84^ ^,^ ^85^, such as, for example, the one by Pendleton et al.^86^ who used tetrahydro curcumin (THCUR), the most bioavailable metabolite of curcumin, together with 5-FU in three squamous cells esophageal cancer (SCC) cell lines, TE-1, TE-8, and KY-5. These cell lines have varying percentages of Cancer Stem Cells (CSC) (totipotent). The researchers observed that THCUR was more effective than 5-FU in all three strains; in the KY-5 strain, with the highest percentage of CSC, THCUR had the greatest effect, THCUR at a dosage of 40µM (above the IC50) in combination with 5-FU significantly suppressed TE-1 cell proliferation, but 5-FU alone did not; and conclude that curcumin does not suppress the effectiveness of 5-FU when supplemented. Despite these findings, in the last five years, no clinical trials with curcumin have been published in this population, which seems to be a powerful field to be explored.

On the other hand, studies that use curcumin in nano-micelles or nanoparticles that carry drugs used in the therapy of esophageal cancer, show excellent results in terms of stability, encapsulation rate, safety, biocompatibility, and better response to suppress tumor growth^87^ ^,^ ^88^. In the future, the use of curcumin as a carrier of drugs used in anticancer therapies, based on the use of pharmaceutical technologies, may become something very common, according to recent publications that show success in its use for this purpose, and may improve tumor responses. and decrease adverse events that are so frequent, which worsen the quality of life and nutritional status of patients^28^ ^,^ ^89^ ^-^ ^91^.

The use of curcumin or curcumin associated with multiple nanoformulations (micelles, polymers, polymer nanocapsules, liposomes, nano gels, gold nanoparticles, etc.), has shown many positive results, inhibiting different signaling pathways related to cell proliferation and stimulating apoptosis pathways^90^ ^,^ ^92^, for example, regulating Beclin1 expression and inhibiting the glycolytic pathway mediated by hypoxia-inducible factor-1α (HIF-1α), involved in the proliferation of pancreatic cancer cells^93^; or by inhibiting the Gli1-β-catenin pathway in gastric cancer cells, the pathway responsible for migration, invasion and remodeling of the cell cytoskeleton.

Overall, curcumin has shown an antitumor and chemoresistance-suppressing effect in all types of gastrointestinal cancers, including metastatic ones. Currently, no studies have investigated curcumin supplementation in sarcopenic patients with gastrointestinal cancer, highlighting numerous opportunities for future research in this population.

In other types of cancer, such as breast cancer, for example, the use of turmeric, especially in conjunction with chemotherapy treatment with tamoxifen, is discouraged due to the lack of evidence^94^ ^-^ ^96^. However, there is a wide path to be explored in clinical practice, making studies with well-designed human beings necessary.

## CONCLUSION

Although premature mortality rates are expected to decrease in Brazil between 2026-2030, as discussed earlier in this review, cancer management, with or without sarcopenia, remains challenging and requires actions at both the public health level and through multimodal interventions in institutions serving this population. Nutrition is a key element in the care of cancer patients, encompassing early detection of nutritional risk and sarcopenia, nutritional intervention, management of gastrointestinal symptoms, and provision of adequate calories, proteins, and other nutrients that help maintain or recover body composition, such as omega-3 fatty acids. 

Curcumin supplementation appears to be a promising adjuvant to standard cancer treatment, helping to reduce chemotherapy side effects, improve tumor response to treatment, and assist in managing sarcopenia by reducing muscle depletion and oxidative stress, and improving strength and fatigue. However, further clinical studies are still needed to understand its benefits in this regard fully.
